# Vision-Based Localization Method for Picking Points in Tea-Harvesting Robots

**DOI:** 10.3390/s24216777

**Published:** 2024-10-22

**Authors:** Jingwen Yang, Xin Li, Xin Wang, Leiyang Fu, Shaowen Li

**Affiliations:** Key Laboratory of Agricultural Sensors, Ministry of Agriculture and Rural Affairs, School of Information and Artificial Intelligence, Anhui Agricultural University, Hefei 230036, China; yjw1849609276@163.com (J.Y.); lixinsaq2001@163.com (X.L.); kingxin_wx@163.com (X.W.); fly2008@ahau.edu.cn (L.F.)

**Keywords:** deep learning, RGB-D, tea, picking point localization

## Abstract

To address the issue of accurately recognizing and locating picking points for tea-picking robots in unstructured environments, a visual positioning method based on RGB-D information fusion is proposed. First, an improved T-YOLOv8n model is proposed, which improves detection and segmentation performance across multi-scale scenes through network architecture and loss function optimizations. In the far-view test set, the detection accuracy of tea buds reached 80.8%; for the near-view test set, the mAP_0.5_ values for tea stem detection in bounding boxes and masks reached 93.6% and 93.7%, respectively, showing improvements of 9.1% and 14.1% over the baseline model. Secondly, a layered visual servoing strategy for near and far views was designed, integrating the RealSense depth sensor with robotic arm cooperation. This strategy identifies the region of interest (ROI) of the tea bud in the far view and fuses the stem mask information with depth data to calculate the three-dimensional coordinates of the picking point. The experiments show that this method achieved a picking point localization success rate of 86.4%, with a mean depth measurement error of 1.43 mm. The proposed method improves the accuracy of picking point recognition and reduces depth information fluctuations, providing technical support for the intelligent and rapid picking of premium tea.

## 1. Introduction

Tea is rich in catechins, theaflavins, and other health-promoting compounds, resulting in an increasing demand for high-quality tea among consumers [1]. However, the harvesting of premium tea still primarily relies on manual labor, and with the rapid development of industrialization and urbanization, issues such as agricultural labor shortages and rising costs are becoming increasingly prominent. This situation highlights the urgency and necessity of developing intelligent tea-picking robots.

In the tea-picking robot system, the vision module plays a critical role, with its core task being the precise identification of tea buds and localization of picking points in the tea stem area in complex tea garden environments [2]. Existing robot vision system designs primarily involve image processing methods and deep-learning-based approaches. Image processing techniques rely on physical characteristics (such as shape, color, and texture) to identify and segment tea buds [3], but they are highly dependent on environmental factors and have limited generalization ability, restricting their use in complex tea garden environments. Deep learning methods, due to their superior feature extraction and generalization abilities, demonstrate significant advantages in visual perception tasks within complex agricultural environments [4,5,6,7,8,9,10,11]. Chen et al. [12] successfully implemented the Faster R-CNN model for tea bud recognition and employed the fully convolutional network (FCN) to segment the tender stem area and locate the picking points. The average accuracy of picking point identification reached 84.91%. Meng et al. [13] combined the improved YOLOX-tiny model with the PSP-net model to achieve bud ROI detection and tea stem area segmentation during the inference stage, improving the picking point localization accuracy to 95.6%. Li et al. [14] proposed a lightweight Yolov5s-TB model, which ensures real-time performance while simplifying the post-processing by extracting the coordinate mean of the bud target’s stem position as the picking point. So far, most research has concentrated on the 2D detection and localization of tea targets in specific scenarios [8,15,16], while exploration of picking point localization in 3D space has been relatively limited.

The key to achieving accurate picking point localization in 3D space lies in obtaining high-quality depth information. In recent years, with the popularization of consumer-grade RGB-D cameras, 3D localization methods based on RGB-D information have gradually become a research hotspot [17,18,19,20,21,22]. Li et al. [23] proposed an innovative method, which first uses the Yolov3 algorithm for bounding box localization of tea bud regions, followed by a clustering algorithm to separate the background point cloud information, and finally uses a cylindrical envelope method to derive the 3D coordinates of the picking point, achieving a good balance between accuracy and real-time performance. However, the effectiveness of point cloud pre-processing and clustering depends on the quality of the depth map, and when the depth map quality is poor, the accuracy of clustering and filtering is affected. Chen et al. [24] proposed a method for picking point localization that combines RGB-D cameras, the YOLO-v3 algorithm, skeleton extraction, and a minimum bounding rectangle, achieving an average depth localization error of 4.2 mm and a tea-picking point accuracy rate of 83%. However, this method still depends on traditional image processing techniques during the post-processing stage, resulting in poor robustness in real field environments, and the localization accuracy is limited by the camera’s position. These studies indicate that the perception quality of depth cameras is rather limited at long distances, especially in complex backgrounds, affecting the accuracy of picking point localization [25,26,27,28,29].

Accurately localizing the picking point requires a comprehensive solution to the two key problems of target recognition and accurate acquisition of depth information. Under the complex unstructured background, improving the recognition segmentation accuracy of tea buds and stems is crucial for building an efficient and reliable vision system for tea-picking robots. In addition, limited by the depth camera accuracy, the precision visual localization of tea stems at distant locations is achieved with low accuracy and high error, which further limits the accuracy of picking point localization. Existing studies have shown that hand–eye coordination control based on RGB-D sensors can effectively improve the operational efficiency of picking robots. By using a “see and move” approach [30,31,32], combining rough localization at long distances with fine localization at close range, the issue of insufficient depth information from a single perspective can be effectively mitigated.

To address the above problems, this study proposes a vision-based localization method that integrates RGB-D information. By building a T-YOLOv8n model, the tea buds and tea stems’ ROI and masks are output accurately and quickly, laying the groundwork for visual perception and designing a layered visual servoing localization strategy for picking points, where coarse localization of tea buds in far views guides fine localization of tea stems in near views, effectively addressing the depth information error issue and achieving 3D localization of the picking point, providing theoretical support for autonomous picking applications.

## 2. Materials and Data

### 2.1. Premium Tea-Picking Standards

**Figure 1:**
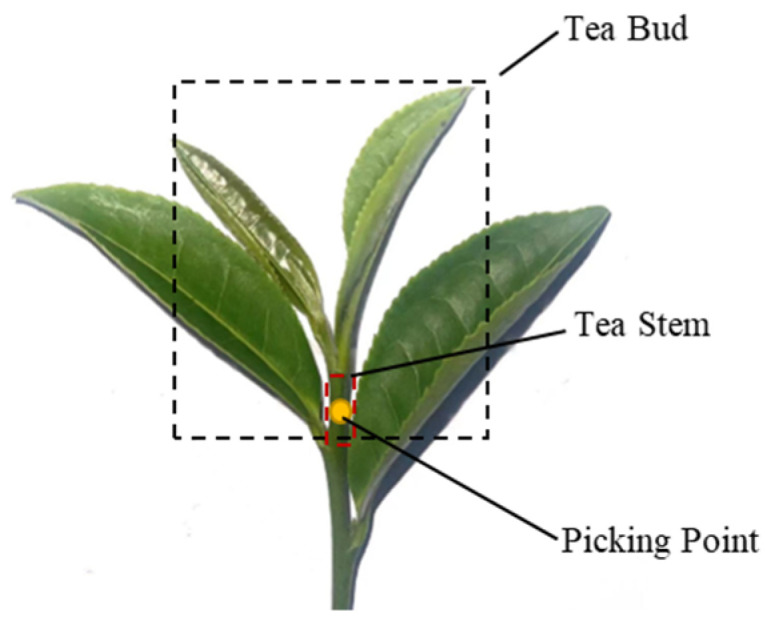
Picking operation point positioning principle.

This study adopts the “one bud and two leaves” standard, which is in high demand, as the basis for discussion, as shown in Figure 1. Manual harvesting is performed using the “pull-picking” method to separate the tea, with the picking point located at the tea stem (potential plucking area). To achieve precise picking point localization based on this mechanism, it is necessary to identify standard-compliant tea buds and accurately segment the stem pixel area. Therefore, in this study, tea buds and stems are defined as the key structural features required for picking.

### 2.2. Data Collection and Construction

Tea samples were collected from the Central Anhui Experimental Station of Anhui Agricultural University, located in Lujiang County, Anhui Province, focusing on the Longjing 43 and Baihaozao varieties.

**Figure 2:**
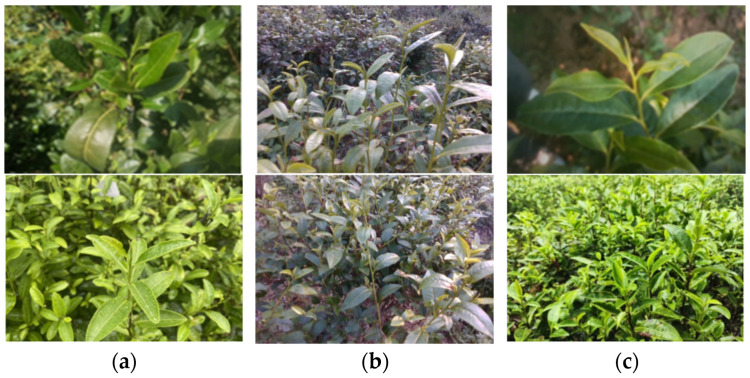
Tea samples in different scenarios. (**a**) Complex lighting: different lighting conditions (e.g., sunny and cloudy days); (**b**) multi-angle observation: images captured from 30° and 60° angles; (**c**) background complexity: varying levels of background clutter.

To build a training dataset for the visual model, RGB images of tea were captured using various devices at different times. To enhance the robustness of the visual localization algorithm, data collection was conducted under diverse environmental conditions, including variations in time, weather, background complexity, and depth of field, with special attention given to the viewpoint requirements for robotic arm operations. The optical axis of the camera was positioned at an angle between 30° and 60° to the horizontal plane, without any tilt along the horizontal axis, to ensure the clarity of the tea stems within the field of view. Considering that in real tea-picking scenarios the robot needs to handle multi-scale dynamic field-of-view information from distant to close ranges, a dynamic collection strategy ranging from far to near (15 cm to 60 cm) was employed to improve the model’s adaptability in practical applications, covering multiple shooting scenes, as illustrated in Figure 2. For localization testing, the Intel RealSense D435i depth camera (hereafter referred to as D435i) was used to collect RGB-D images as test samples. The device can simultaneously output both RGB images and depth maps, supporting a frame rate of up to 90 FPS. Since the RGB and depth images come from different sources, image registration was required to transform the depth image coordinate system into the RGB image coordinate system, ensuring that each pixel’s depth value corresponded accurately.

After filtering, a total of 2109 valid images were obtained for model training, which were divided into a training set (1687 images) and a validation set (422 images) in an 8:2 ratio. The Labelme [33] software (version 4.5.13) was used for annotation, with rectangular boxes labeled “Tea” (tea buds) and polygons labeled “Stem” (tea stems) for the two target classes.

**Figure 3:**
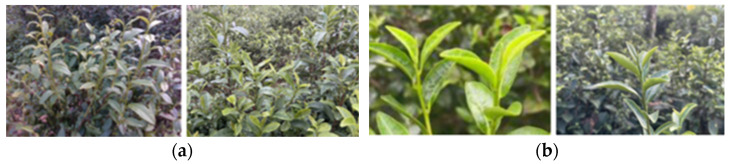
Construction of MFN combined test sets: (**a**) test set A; (**b**) test set B.

In addition, an independent combined MFN (Multi-Focus Near and Far) test set was constructed to comprehensively evaluate the model’s performance. It includes far-view test set A (170 images) and near-view test set B (150 images). Far-view test set A is characterized by multiple targets (≥4), complex backgrounds, and diverse lighting conditions; near-view test set B is characterized by fewer targets (1–3), simple backgrounds, and clearly visible tea stems. The composition of the dataset is shown in Table 1, and examples of MFN are shown in Figure 3.

## 3. Methods

### 3.1. T-YOLOv8n: A Tea-Picking Key Structure Recognition and Segmentation Model Based on an Improved YOLOv8n-Seg

YOLOv8 is an algorithm framework developed and maintained by the Ultralytics team [34], featuring broad adaptability and supporting a variety of task demands, including object detection, instance segmentation, and pose estimation [35,36]. YOLOv8n-segment is an extended single-stage instance segmentation algorithm based on YOLOv8, with multi-task learning capabilities that efficiently perform both object detection and instance segmentation tasks simultaneously. In the task of picking point localization, the model can quickly locate the region of interest (ROI) of tea buds and precisely segment the tea stem, extracting the necessary key structural features, thereby offering essential visual perception support for precise picking.

**Figure 4:**
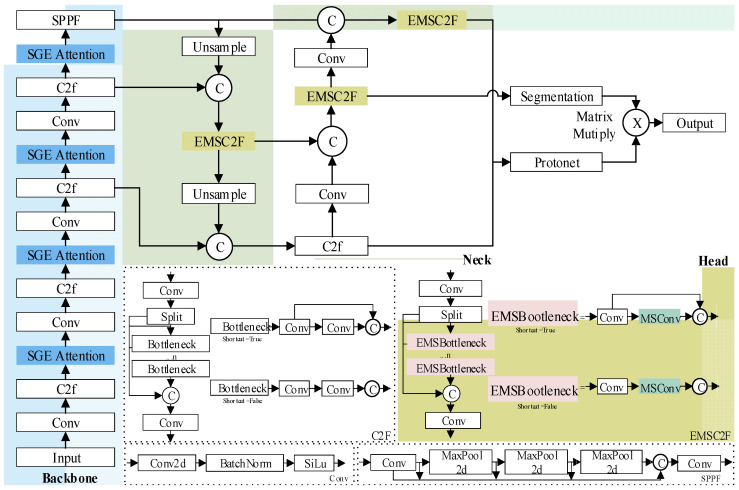
T-YOLOv8n model framework diagram (improvements are marked in color).

However, the complexity of the tea garden environment poses challenges for the model application, including varying backgrounds, densely distributed targets, and diverse lighting conditions. Moreover, tea-picking robots need to handle dynamic, multi-scale information flows [37], which place higher demands on the model’s real-time processing and generalization capabilities. To address these challenges, this study structurally optimized the YOLOv8n-seg model, aiming to enhance its generalization and robustness across multi-scene and multi-scale views. The improved model is named Tea-YOLOv8 nano (abbreviated as T-YOLOv8n), and its structure is shown in Figure 4.

The model architecture is mainly composed of four parts: input, backbone, neck, and head. First, the backbone is responsible for feature extraction from the input image. The SGE attention module is inserted after each convolutional layer in the backbone, allowing the model to focus more on key structural features related to the picking task (e.g., tea buds and stems), effectively enhancing the feature extraction capability of the backbone network. Next, the extracted feature information is passed through the neck for feature fusion. By introducing the EMSC2F module to replace the original C2F module, the model can better fuse features at different levels, thus capturing both local details and global contextual information. The head portion uses a decoupled head structure, with separate branches learning object position regression and classification information, improving the efficiency of multi-task learning. Additionally, the model employs an anchor-free design, which directly fits object size by learning the boundary distances and key point positions, reducing reliance on anchor boxes and better adapting to the requirements of object detection and segmentation at different scales. In Figure 4, SGE attention represents the SGE attention mechanism module, and EMSC2F represents the enhanced multi-scale feature fusion module.

#### 3.1.1. Feature-Enhanced Backbone Network

In complex tea garden environments, the original backbone network tends to extract target and background features without discrimination. To improve the model’s ability to extract key features, an SGE attention mechanism (spatial group-wise enhance attention) module [38] was introduced after each convolution operation. The SGE module enhances key features locally and suppresses redundant background information, significantly improving the model’s feature representation capability. By fusing attention features at different scales, the improved backbone network generates a feature map with rich multi-scale contextual information, enhancing the model’s feature extraction ability in multi-scene, multi-view tea garden environments, thus improving the model’s overall detection and segmentation accuracy.

**Figure 5:**
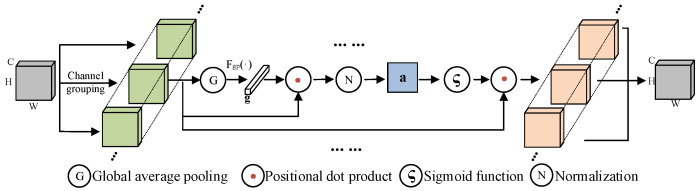
SGE working principle diagram.

The core idea of the SGE module is to introduce a spatial group-wise enhancement mechanism that selectively enhances feature expressions at different spatial locations. Specifically, the SGE module divides the input feature map along the channel dimension into several sub-feature groups, and each sub-feature group undergoes independent attention computation. This grouping strategy enables the model to generate attention weights based on different spatial locations, dynamically adjusting the importance of each sub-feature, enhancing the expression of key features while suppressing potential background noise. The SGE workflow is shown in Figure 5, and the steps are as follows below.

Step 1 (Input feature grouping): The input feature maps (dimensions C × H × W) are divided along the channel into sub-feature groups, where C represents the number of channels, and H and W represent the height and width of the feature map, respectively. Each sub-feature group can be represented by a feature vector: $x_{i}\in R^{\frac{C}{A}}$, *i* = *1*, *2*, …*m*, *m* = *H* × *W*.

Step 2 (Global average pooling): apply global average pooling to each sub-feature group to obtain the specific semantic feature representation *g* ∈ $R^{\frac{C}{A}}$ for the group, as shown in Equation (1):

(1) $$g=\frac{1}{m}\sum_{i=1}^{m}x_{i}$$

Step 3 (Attention coefficient calculation and normalization): The attention coefficient $c_{i}$ is computed by performing a point-wise dot product between the global feature g and the local feature $x_{i}$ of each sub-feature group. The attention coefficient is then normalized (N) to obtain the normalized attention coefficient $\bar{c_{1}}$, as shown in Equation (2). Here, $\mu_{c}$ and $\sigma_{c}$ represent the mean and standard deviation of the attention coefficient.

(2) $$\bar{c_{1}}=\frac{c_{i}-\mu_{c}}{\sigma_{c}+\varepsilon }=\frac{g\times x_{i}-\mu_{c}}{\sigma_{c}+\varepsilon }$$

Step 4 (Attention weight generation): To ensure that the SGE module can achieve identity transformation, learnable parameters *γ* and *β* are introduced to scale and shift the normalized attention coefficient. The transformed attention coefficient $c_{1}$ is then passed through a Sigmoid activation function to generate the final attention weight $a_{i}$, as shown in Equations (3) and (4):

(3) $$\bar{c_{1}^{\prime }}=\gamma \bar{c_{1}}+\beta$$

(4) $$a_{i}=\sigma \times \bar{c_{1}^{\prime }}=\sigma \times \left(\gamma \bar{c_{1}}+\beta \right)$$

Step 5 (Feature enhancement): The SGE module multiplies the original feature $x_{i}$ elementwise by the attention weight $a_{i}$, resulting in the enhanced feature vector $\bar{x_{i}}$, which selectively enhances key structural features such as tea buds and stems in the spatial dimension, filtering out redundant background information.

#### 3.1.2. EMSC2F Feature Fusion Module 

To address the issue of single-scale C2F (CSPDarknet53 to 2-Stage Feature Pyramid Network) module’s insufficient sensitivity to the morphological features of tea buds and stems, a lightweight EMSC2F (Enhanced Multi-Scale Convolution to 2-Stage Feature Pyramid Network) module is proposed. This module introduces MSConv (Multi-Scale Convolution) operations, effectively capturing multi-scale features while maintaining the same number of parameters and computational complexity. It enhances attention to multi-scale information, thereby improving the perception of small objects.

**Figure 6:**
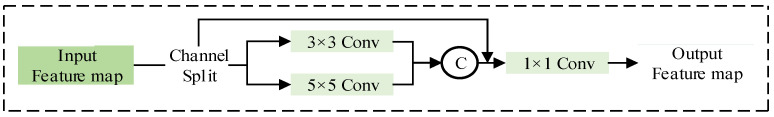
MSConv convolution structure.

The MSConv convolution module (as shown in Figure 6) draws on the design concept of GhostNet [39], aiming to reduce the parameter count and computational cost of standard convolution operations while maintaining performance. The working principle is as follows: First, the input features are divided into two parts along the channel dimension. One part is retained as a shallow feature branch to preserve key information integrity, while the other part, as a multi-scale feature branch, is further divided into two sub-groups, processed by 3 × 3 and 5 × 5 convolution kernels, respectively. These processed features are rearranged along the channel dimension and concatenated with the shallow features, and finally, a 1 × 1 pointwise convolution [40] is used to fuse the channels.

Specifically, some C2F modules in the neck network of YOLOv8n-seg were improved to form the EMSC2F module. In the bottleneck structure of the C2F module, the MSConv convolution module was introduced, replacing the original second convolution layer to form the EMSBottleneck. This improvement allows the EMSBottleneck to more effectively handle the output features generated by the first convolution, thereby enhancing feature fusion.

#### 3.1.3. Scale-Adaptive Loss Function

The original YOLOv8n-seg uses the CIoU (complete intersection over union) [41] loss function. CIoU takes into account the intersection over union (IoU) between the predicted and ground truth boxes, the distance between their center points, and aspect ratio consistency. The CIoU loss function is defined in Equation (5):

(5) $$L_{CIoU}=1-CIoU=1-\left(IoU-\frac{\rho^{2}\left(b,b^{gt}\right)}{c^{2}}+\alpha v\right)$$

(6) $$v=\frac{4}{\pi^{2}}\left(arctan\frac{w^{gt}}{h^{gt}}-arctan\frac{w}{h}\right)$$

where IoU represents the intersection over union between the predicted and ground truth boxes; b, $b^{gt}$ represents the center point coordinates of both boxes; ρ($b,b^{gt}$) is the Euclidean distance between the center points; *c* is the diagonal length of the smallest enclosing box; α is the weight coefficient; and *v* measures the aspect ratio consistency penalty.

However, CIoU mainly measures the relative aspect ratio and neglects the absolute size differences of the target. In the tea garden environment, due to the significant morphological differences in tea buds and stems, the target size varies greatly, which can result in cases where the predicted and ground truth boxes have the same aspect ratio but different absolute sizes. In such cases, the aspect ratio penalty term v in CIoU becomes ineffective and fails to optimize the bounding boxes properly. Moreover, CIoU’s fixed penalty mechanism performs limitedly when handling low-quality samples, making it difficult to learn useful information and thus affecting the overall detection accuracy. To address these issues, we introduced the Powerful-IoU v2 (PIoUv2) [42] loss function, which optimizes the model’s convergence speed and improves segmentation accuracy for small targets. PIoUv2 further optimizes PIoU by adding a penalty factor P based on the boundary distance between the predicted and ground truth boxes. It adaptively adjusts the loss weights for different target sizes by calculating the differences between the left, right, top, and bottom boundaries of the predicted and ground truth boxes. This mechanism enhances the model’s ability to perceive the position and shape of the predicted boxes, especially excelling in handling complex shapes and small objects.

Additionally, PIoUv2 introduces a dynamic adjustment mechanism that adaptively adjusts the gradient based on anchor box quality, using the dynamic penalty term $e^{-p}$, which adaptively adjusts the loss weight based on the quality of the anchor box. For low-quality anchor boxes (i.e., predicted boxes that deviate significantly from the ground truth), PIoUv2 generates smaller gradients, gradually guiding the predicted boxes toward the ground truth boxes and avoiding over-adjustment. For medium-quality anchor boxes, PIoUv2 applies larger gradients, prompting the model to converge more quickly to high-quality predicted boxes. The boundary distance penalty and dynamic adjustment mechanism of PIoUv2 allow the model to progressively refine the edges and complex details of the target, improving the accuracy of edge detection and segmentation.

The mathematical expression of the PIoUv2 loss function is as follows:

(7) $$L_{PIoUv2}=3\cdot \lambda e^{-p}{\cdot e}^{{-\left(\lambda e^{-p}\right)}^{2}}\cdot L_{PIoU}$$

In Equation (7), *λ* is a hyperparameter, and $e^{-p}$ is the penalty term of PIoUv2. The $L_{IoU}$ consists of two parts the original IoU loss $L_{IoU}$ and the penalty term $R_{PIoU}$. The specific formula is as follows:

(8) $$P=\frac{dw_{1}}{w_{gt}}+\frac{dw_{2}}{w_{gt}}+\frac{dh_{1}}{h_{gt}}+\frac{dh_{2}}{h_{gt}}$$

(9) $$L_{PIoU}=L_{IoU}+R_{PIoU}=L_{IoU}+1-e^{{-P}^{2}}$$

**Figure 7:**
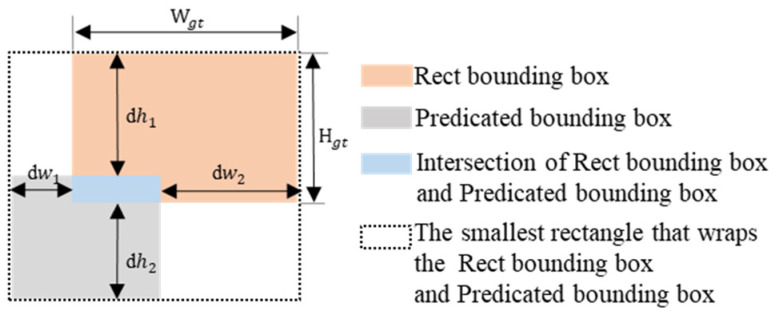
PIoU loss function.

Equation (9) is the PIoU loss function formula, where *P* is the scale-adaptive penalty factor for PIoU targets, $w_{gt}$ and $h_{gt}$ represent the width and height of the ground truth box, and $dw_{1}$, $dw_{2}$, $dh_{1}$, and $dh_{2}$ represent the distances between the ground truth boxes, as shown in Figure 7.

### 3.2. A Layered Visual Servoing Strategy for Picking Point Localization Integrating Far and near Views

In unstructured tea garden environments, complex backgrounds and slender tea stems pose challenges for robotic visual perception. As a critical component in the tea-picking process, the positioning accuracy of the tea stems directly affects the success rate of the picking operation. However, in practical applications, due to the limitations in depth camera resolution and depth information acquisition capabilities, the single-stage visual identification of slender tea stems results in significant positioning errors.

**Figure 8:**
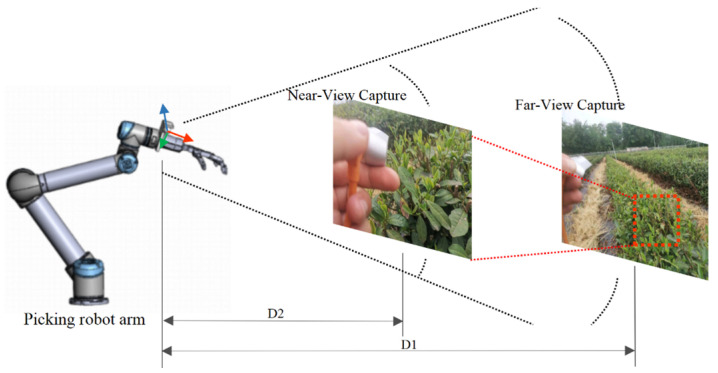
Example of layered visual spatial localization strategy for picking points in near and far views.

To address this issue, this study proposes a visual servoing localization strategy for picking points based on a near–far view stratification. The core idea of this strategy is to adjust the posture of the robotic arm and the relative position between the camera and the target to obtain image data at different detection distances and to achieve 3D localization of the picking point using the output of the T-YOLOv8n model proposed in Section 3.1. In the far-view scenario, since the perception range is wide, but the detailed information of the target is limited, the T-YOLOv8n model can effectively recognize the region of interest (ROI) of the tea buds. In the near-view scenario, by narrowing the field of view, the detailed information of the target is enhanced, while the precision and accuracy of depth perception are improved, leading to the precise segmentation of the tea tip contours. With this near–far view stratification method, combining coarse localization in the far view and fine localization in the near view, this strategy ensures high detection and segmentation performance at varying distances while maintaining the accuracy and reliability of the picking point localization. Figure 8 shows an example of this strategy.

**Figure 9:**
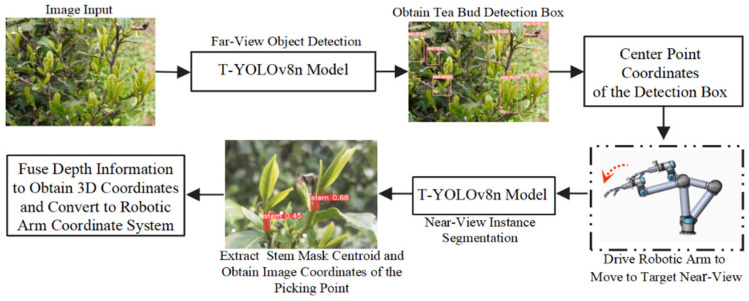
Flowchart for spatial localization of picking points.

In Figure 8, D1 represents the far-view shooting position (with the camera approximately 400–600 mm away from the tea canopy), while D2 represents the near-view shooting position (with the camera approximately 200–300 mm away from the tea canopy). The setup of these distances is based on a comprehensive consideration of various factors, including camera imaging quality, depth measurement accuracy, and the limitations of the robot’s working space. First, the purpose of D1 is to achieve rough target localization by expanding the field of view, while ensuring that the image resolution is sufficient to detect a larger target area. In contrast, D2 is primarily used to improve the accuracy of depth information acquisition. Within its set range, it effectively reduces errors, enabling precise target detection and segmentation. By properly setting D1 and D2, the system ensures both the perceptual range and the acquisition of high-resolution images of the target area. The spatial localization process of the picking point based on the far–near layered visual servoing strategy is shown in Figure 9, and the specific steps are as follows below.

Step 1 (Far-view Target Recognition): At the D1 position in the far view, the RGB-D camera captures a far-view color image. The T-YOLOv8n model is utilized to quickly detect tea bud ROIs from the complex background, preliminarily determining the target’s depth information and potential picking point position.

Step 2 (Near-view Picking Point Localization): Based on the far-view recognition results, the robotic arm is guided to move to the near-view shooting position D2 for each tea target in sequence. Using near-view high-resolution images, the fine structural features of stems are extracted [20], achieving pixel-level precise segmentation of the tea stems. The centroid moment [43] method (Equation (10)) is used to calculate the centroid of the segmented mask, which corresponds to the pixel coordinates of the picking point ($x_{c},y_{c}$). Combined with the depth value $Z_{c}$ obtained from the camera’s intrinsic parameters [44], the corresponding depth value for the pixel coordinates is matched, thus determining the 3D coordinates of the picking point *P* ($x_{c},y_{c},Z_{c}$).

(10) $$x_{c}=\frac{\sum_{i=1}^{n}x_{i}f\left(x_{i},y_{i}\right)}{\sum_{i=1}^{n}f\left(x_{i},y_{i}\right)},\;y_{c}=\frac{\sum_{i=1}^{n}y_{i}f\left(x_{i},y_{i}\right)}{\sum_{i=1}^{n}\left(x_{i},y_{i}\right)}$$

Step 3 (Picking Execution): The three-dimensional coordinates ($x_{c},y_{c},Z_{c}$) of the picking point P are transformed from the camera coordinate system to the robot base coordinate system, thereby determining the absolute position ($x_{r},y_{r},Z_{r}$) of the picking point in the robot’s workspace and then controlling the robotic arm to execute the picking.

To ensure the efficient application of this strategy with the RGB-D camera, the T-YOLOv8n model performs scaling and padding on the input images during the inference stage to accommodate different image sizes, thus enabling the comprehensive deployment of the layered near and far view perception strategy.

## 4. Experiments and Results

### 4.1. T-YOLOv8n Performance Experiments

#### 4.1.1. Experimental Setup and Evaluation Indexes

**Figure 10:**
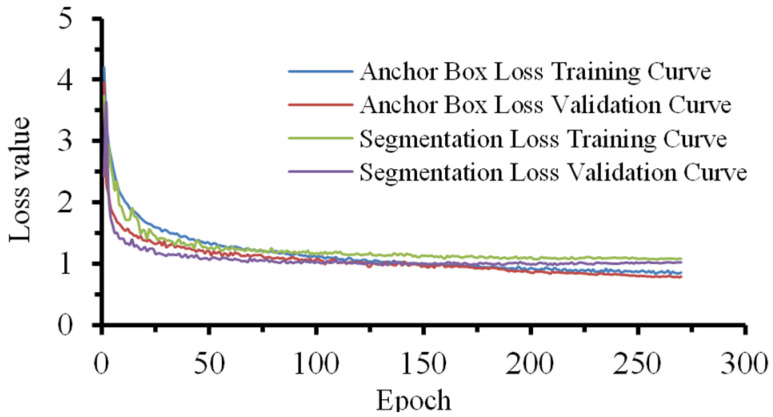
Model training loss curve.

This study conducted experiments using the Pytorch framework on the Ubuntu 20.04 operating system. The hardware configuration includes an NVIDIA RTX 4090 Ti GPU (24 GB VRAM) and a 13th Gen Intel(R) Core (TM) i7-13700 processor. The experimental parameters are set as follows: input image resolution is 640 × 640 pixels, batch size is 16, and the training cycle is 300 epochs. The initial learning rate is set to 0.01, and the optimizer uses the stochastic gradient descent (SGD) algorithm. Figure 10 shows the model’s training process. The training loss and validation loss of the model rapidly decrease as the number of training epochs increases, indicating that the model is effectively learning from the data. As training progresses, the loss curves eventually flatten out, reflecting the model’s fast convergence and the gradual stabilization of the training process. The continued decrease in training loss indicates that the model effectively reduces training errors, while the simultaneous decrease in validation loss suggests that the model demonstrates good generalization ability on the validation set.

To comprehensively evaluate the performance of the T-YOLOv8n model under different fields of view and tasks, a composite test set (MFN) is used for targeted evaluation. Test set A is used to evaluate the model’s performance in identifying tea buds within a larger field of view. Test set B evaluates the model’s recognition and segmentation performance for tea stems. To verify the model’s effectiveness, precision (P), recall (R), and mean average precision (mAP) are selected as the accuracy evaluation metrics. Additionally, the model’s parameter count, number of floating-point operations, and frames per second (FPS) are also recorded as efficiency evaluation metrics.

#### 4.1.2. Ablation Experiment

To validate the effectiveness of the improvement strategies in the T-YOLOv8n model, ablation experiments were conducted on the different optimization strategies mentioned in Section 3.1, with YOLOv8n-seg as the baseline model. The results are shown in Table 2.

In the tea bud detection task of test set A, the baseline model’s precision, recall, and mAP_0.5_ are 74.6%, 74.8%, and 74.6%, respectively. After introducing the PIoUv2 loss function, the mAP_0.5_ increased to 78.1%, a 3.5% improvement over the baseline model. At the same time, precision and recall increased by 4% and 2.9%, respectively, verifying the effectiveness of the PIoUv2 loss function in improving model localization accuracy. Furthermore, after introducing the EMSC2F module into the neck structure, the model’s recall and mAP_0.5_ improved by 2.3% and 3.6%, respectively, while model parameters were reduced by approximately 4%. This suggests that the module improves performance while reducing model complexity. After introducing the SGE module, the model’s detection precision, recall, and mAP_0.5_ improved by 3.8%, 1.7%, and 4.3%, respectively, confirming that the restructured backbone network has stronger feature extraction capabilities.

In the stem detection and segmentation task of test set B, the baseline model’s bounding box precision, recall, and mAP_0.5_ were 88.5%, 77.3%, and 84.5%, respectively, and the mAP_0.5_ for tea stem mask segmentation reached 79.6%. The introduction of the PIoUv2 loss function increased the mask mAP_0.5_ by 6.5%, effectively improving the segmentation accuracy for small objects. After introducing the EMSC2F module, the accuracy and mAP_0.5_ for stem bounding box recognition increased by 0.9% and 3.4%, respectively, while the accuracy and mAP_0.5_ for tea stem mask segmentation improved by 4.9% and 1.2% compared to the original model. This demonstrates that the module enriches feature representation by integrating multi-scale features, thereby improving the model’s detection and segmentation performance. Additionally, the introduction of the SGE module increased the mask mAP_0.5_ by 4.5% compared to the baseline model, further validating the enhanced backbone network’s superior performance in extracting fine features of tea stems, thereby improving overall accuracy. Meanwhile, the improved model reduces the parameter count and GFLOPS by 3% and 5.8%, respectively, compared to the baseline model, making it more advantageous for resource-constrained mobile device and robotics applications.

**Figure 11:**
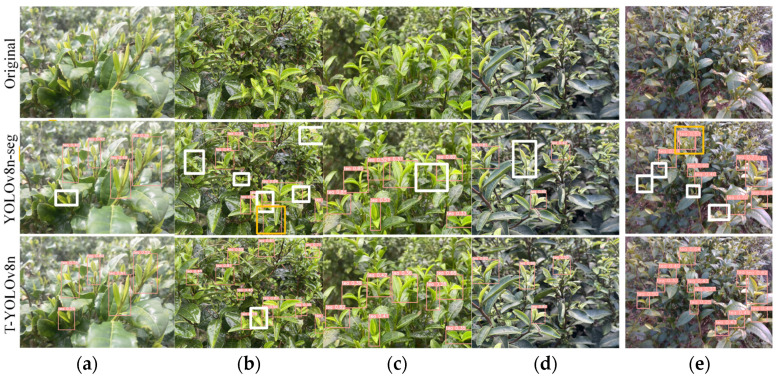
Detection results of tea buds (tea label) in test set A. (**a**) Strong light; (**b**) rainy; (**c**) normal light + 30° angle; (**d**) normal light + 45° angle; (**e**) normal light + 60° angle. Note: white boxes indicate missed detections, and yellow boxes indicate false detections.

Figure 11 compares the bud detection performance of the baseline model and T-YOLOv8n in test set A. The figure shows the performance of T-YOLOv8n under various lighting conditions such as strong light, rainy days, and normal lighting, reflecting the model’s adaptability to changing lighting environments. Additionally, the test includes images captured from 30°, 45°, and 60° angles to simulate the typical viewpoints of the robotic arm during real-world operations. When comparing the strong light (a) and rainy day (b) scenarios, the baseline model exhibited missed detections in both cases, especially in the rainy-day scenario where dense target distribution also led to false detections. In contrast, the improved T-YOLOv8n performed significantly better in these scenarios. In the normal lighting scenes with 30° (c), 45° (d), and 60° (e) viewing angles, the baseline model still showed varying degrees of missed detections, especially in the (e) scenario where the complex background and dense target distribution led to a higher miss rate in small object detection. In contrast, T-YOLOv8n showed significantly improved detection performance in this scenario. Overall, compared to the baseline model, T-YOLOv8n significantly reduced missed and false detections, demonstrating stronger environmental adaptability and robustness.

**Figure 12:**
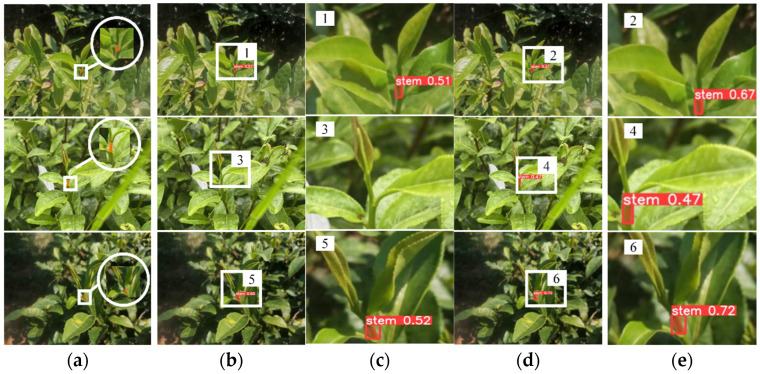
Segmentation effects for stem recognition in test set B. (**a**) Original image and annotation information (the area within the white circle is a magnified view of the region outlined by the square); (**b**) YOLOv8n-seg; (**c**) local enlargement (localized enlargement of the area within the rectangular box corresponding to (**b**)); (**d**) T-YOLOV8n; (**e**) local enlargement (localized enlargement of the area within the rectangular box corresponding to (**d**)).

Figure 12 further compares the tea stem recognition and segmentation performance of the baseline model and T-YOLOv8n in test set B. The test scenes included complex lighting conditions such as uneven illumination and shadowed areas. T-YOLOv8n excels in the accuracy of bounding box localization and the completeness of mask segmentation in the tender stem regions, enabling more precise localization of picking points. The improved model demonstrated better robustness under different lighting conditions, further validating its generalization ability in multi-task and multi-scale image scenarios.

#### 4.1.3. Comparative Experiments

To further evaluate the superiority of the T-YOLOv8n model, it was compared with the two-stage instance segmentation model Mask R-CNN [45] and the single-stage instance segmentation models YOLACT [46], YOLOv5n-seg [47], and YOLOv8n-seg in both the far-view test set A and the near-view. The test results are shown in Table 3.

In the tea bud detection task of test set A, the T-YOLOv8n model’s mAP_0.5_ reached 81.0%, representing a 7.9% and 1.3% improvement over YOLOv5n-seg and YOLACT, respectively. In the tea stem segmentation task of the near-view test set B, its mask mAP_0.5_ reached 93.7%, respectively, representing improvements of 9.1% over YOLOv5n-seg. Compared to YOLOv8n-seg, the improvement is 14.1%, while the improvements over YOLACT and over Mask R-CNN are 41.4% and 8.3%, respectively.

Additionally, T-YOLOv8n also has advantages in terms of complexity, weight size, and parameter count. The size of the T-YOLOv8n model is only 6.3 MB, which is much smaller than the YOLACT and Mask R-CNN models, making it more suitable for deployment in resource-constrained environments. T-YOLOv8n achieves 178 frames per second (FPS), which is significantly higher than the 30 FPS of Mask R-CNN, whose accuracy is second-best among the models. T-YOLOv8n maintains high accuracy while achieving faster processing speed, making it more suitable for real-time tasks such as tea-picking robots.

The results show that T-YOLOv8n performs excellently in detection and segmentation tasks across multi-task scenarios and also has advantages in terms of complexity, weight size, and parameter count. This proves that T-YOLOv8n effectively supports the far–near layered visual servoing localization strategy, providing a visual algorithm foundation for the precise perception of tea-picking robots.

### 4.2. Picking Point Localization Experiment

**Figure 13:**
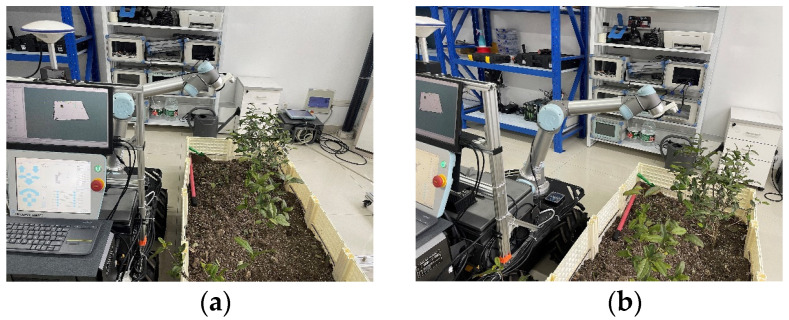
Picking point localization experiment based on a layered near and far view strategy experiments. (**a**) Far-view point; (**b**) near-view point.

To verify the effectiveness of the far–near layered visual servoing in picking point localization, an online comparison experiment was conducted between a single-stage visual scheme and a two-stage visual (far–near layered) scheme. The experimental platform consists of key equipment such as a robotic arm, depth camera, main computer, and mobile platform (as shown in Figure 13). The robotic arm assists the vision system in completing the localization of picking points. The depth camera is used to capture RGB images and depth information, combining with vision algorithms for 3D localization. The mobile platform performs movement operations in subsequent stages.

The experiment includes 20 sets of independent tests, with each set collecting 20 frames of corresponding far and near images from a fixed robotic arm position, including RGB color images and depth maps. The single-stage visual scheme directly identifies and localizes tea stems from the far-view images. The two-stage visual scheme first identifies buds from far-view images and then precisely identifies and localizes tea stems in the corresponding near-view images.

**Figure 14:**
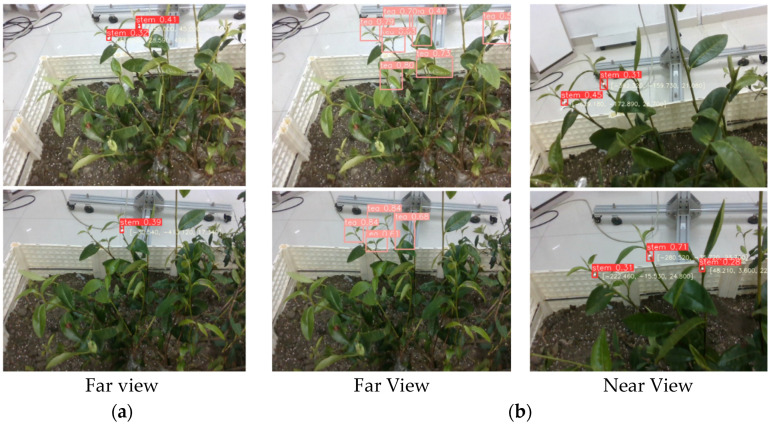
Comparison experiment between single-stage vision and two-stage vision. (**a**) Single-stage visual scheme; (**b**) two-stage visual scheme.

The improved T-YOLOv8n model was used as the visual recognition algorithm during the experiment, and the 3D coordinates of the picking point were calculated by combining the camera’s intrinsic matrix with the depth data. To ensure the spatial mapping relationship between the far- and near-view images and the accuracy of the statistical analysis, only the part of the far-view image corresponding to the near-view image region was analyzed. An example of the experiment is shown in Figure 14.

Table 4 summarizes the experimental results. In the single-stage visual scheme, the far-view tea stem recognition accuracy was 64.4%, and the success rate of picking point localization was only 23.7%. In the two-stage visual scheme, the recognition accuracy of tender buds in far-view images reached 86.6%, while the recognition accuracy of stems in near-view images improved to 88.7%, and the success rate of picking point localization significantly increased to 86.4%. The results indicate that the two-stage visual scheme, combined with the improved T-YOLOv8n model, effectively enhances the recognition accuracy and localization success rate by optimizing near-view fine feature extraction and depth information acquisition.

To quantitatively evaluate the impact of the far–near view strategy on the accuracy of picking point localization, a localization error *Ψ* is introduced, as shown in Equation (11), where *Oz* is the actual depth value of the picking point (measured by a laser rangefinder), and *Pz* is the depth value recorded by the depth camera.

(11) $$\Psi =\left|O_{Z}-P_{Z}\right|$$

**Figure 15:**
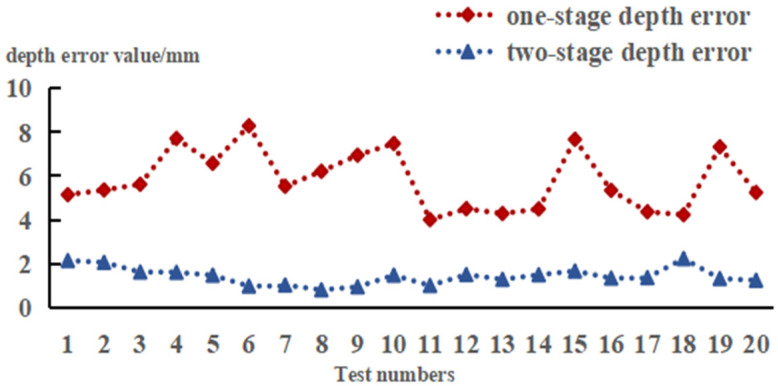
Depth error distribution.

The experimental results are shown in Figure 15. In the single-stage method, the average depth error of successfully localized picking points was approximately 5.8 mm. In the two-stage method, the average depth error was reduced to 1.43 mm, representing a 75.3% reduction in error. Considering that the average diameter of a tea stem is approximately 2 mm (measured manually using a digital caliper), which requires high localization accuracy, the far–near layered strategy effectively reduced fluctuations and errors related to depth information. The localization accuracy of the picking point was improved, meeting the operational requirements of the robot [48].

## 5. Conclusions

This study addresses the challenge of accurate identification and localization of tea-picking robots in unstructured environments and proposes a picking point visual localization method based on RGB-D information fusion. The main contributions include the following:

1. Designed and optimized the T-YOLOv8n model for identifying and segmenting key structures for picking: Based on YOLOv8n-seg, structural optimizations were made, including backbone network reconstruction, feature fusion module design, and the introduction of a scale-adaptive loss function, improving the model’s performance in recognizing and segmenting key tea-picking structures in multi-scale scenarios. In the MFN composite test set, the tea buds in the far-view test set reached 80.8%, and in the near-view test set, the mAP_0.5_ for tea stem detection in bounding boxes and masks reached 93.6% and 93.7%, an improvement of 9.1% and 14.1% compared to the original model, laying a visual perception foundation for accurate picking point localization.
2. Proposed a far–near layered visual servoing strategy: To address the limitations of single-stage visual methods in accurately locating slender tea stems and the inherent shortcomings of low-cost depth cameras in capturing small target information, this study proposes this strategy. This method effectively combines the region of interest (ROI) of tea buds output by the T-YOLOv8n model with the coarse segmentation results of tea stems, enabling accurate localization of the 3D coordinates of picking points.
3. Experimental validation shows that by using this two-stage visual method, the success rate of picking point localization reached 86.4%, with an average depth error of only 1.43 mm, confirming the potential application value of this method in precise robotic operations.

Although this study has made progress in the accuracy of picking point localization, there is still room for improvement. In situations where tea stems are heavily occluded, there are still some cases of missed detection. Future work will further introduce active interaction control of the robotic arm, combining multi-view perception and operation to enhance the robustness of picking point localization in complex unstructured environments [18]. This research presents an effective technical solution for tea-picking robots by employing a layered visual strategy and optimizing the model structure. It specifically addresses the challenges of identifying small targets, such as tea stems, and mitigating the loss of depth information during the localization of picking points. The findings of this study offer critical technical support for the intelligent harvesting of premium tea and establish a solid foundation for further advancements in tea-picking automation.

## Figures and Tables

**Table 1:** Dataset Composition in Detail.

Type of Dataset		Total
Training set		1687
Validation set		422
MFN test set	Far-view test set A	170
	Near-view test set B	150
Total		2429

**Table 2:** Results of ablation experiments.

Dataset (Class)	Add Modules			Bounding Box/%			Mask/%			Parmas/10^6^	GFLOPS
	PIoUv2	EMSC2F	SGE	P	R	mAP_0.5_	P	R	mAP_0.5_		
Test A (Tea)				74.6	74.8	74.6	-	-	-	3.26	12.1
	√			78.6	77.7	78.1	-	-	-	3.26	12.1
		√		73.8	77.1	78.2	-	-	-	3.13	11.4
			√	78.4	76.5	78.9	-	-	-	3.30	11.7
	√	√	√	80.8	76.2	81	-	-	-	3.16	11.4
Test B (Stem)				88.5	77.3	84.5	84.9	71.8	79.6	3.26	12.1
	√			88.6	80.3	88.1	85.8	81.4	86.1	3.26	12.1
		√		89.4	81.7	87.9	89.8	74.6	80.8	3.13	11.4
			√	78.8	78.3	83.3	82.9	82.1	84.1	3.30	11.7
	√	√	√	95.4	88.5	93.6	95.5	89.5	93.7	3.16	11.4

“√” indicates that the module is used.

**Table 3:** Comparative experiment.

Dataset (Class)	Model	Bounding Box/%			Mask/%			Parmas /10^6^	GFLOPS	Model Size /MB	FPS
		P	R	mAP_0.5_	P	R	mAP_0.5_				
Test A (Tea)	YOLOv5n-seg	79.2	72.0	73.1	-	-	-	2.76	11.1	5.9	189
	Mask R-CNN	80.1	82.3	80.3	-	-	-	-	-	53.7	30
	YOLACT	76.3	80	79.7	-	-	-	-	-	45.84	94
	YOLOv8n-seg	74.6	74.8	74.6	-	-	-	3.26	12.1	6.72	151
	T-YOLOv8n	80.8	76.2	81	-	-	-	3.16	11.4	6.3	178
Test B (Stem)	YOLOv5n-seg	91.5	76.3	89.4	86.6	73	84.6	2.76	11.1	5.9	189
	Mask R-CNN	72.3	95.6	84.6	71.1	97.1	85.4	-	-	53.7	30
	YOLACT	70	89.7	68.7	72.3	79.4	52.6	-	-	45.84	94
	YOLOv8n-seg	88.5	77.3	84.5	84.9	71.8	79.6	3.26	12.1	6.72	151
	T-YOLOv8n	95.4	88.5	93.6	95.5	89.5	93.7	3.16	11.4	6.3	178

**Table 4:** Target localization results statistics.

Method	First Target	Second Target	Tea Count (Statistical)	Tea Count (Detected)	Stem Count (Statistical)	Stem Count (Detected)	Number of Successful Picking Poin Localizations
Single-stage visual scheme	Stem	-	-	-	59	38	14
Two-stage visual scheme	Tea	Stem	71	64	59	54	51

In this study, the criterion for successful picking point localization is defined as the picking point being within the identified tea stem area, and valid depth information is successfully obtained for the point.

## Data Availability

The data in this study are available upon request from the corresponding author.
